# The altered expression of cytoskeletal and synaptic remodeling proteins during epilepsy

**DOI:** 10.1515/biol-2022-0595

**Published:** 2023-04-10

**Authors:** Yanbao Xin, Guojiao Lin, Tianbao Hua, Jianmin Liang, Tianmeng Sun, Xuemei Wu

**Affiliations:** Key Laboratory of Organ Regeneration and Transplantation of Ministry of Education, Institute of Immunology, The First Hospital of Jilin University, Changchun 130021, China; The First Hospital and International Center of Future Science, Jilin University, Changchun 130015, China; Department of Pediatric Neurology, The First Hospital of Jilin University, 1 Xinmin Street, Changchun 130000, Jilin Province, China; Jilin Provincial Key Laboratory of Pediatric Neurology, Changchun 130021, China

**Keywords:** neural cytoskeleton, epilepsy, F-actin, neurofilaments, synaptophysin

## Abstract

The cytoskeleton plays an important role in epilepsy; however, the mechanism is unknown. Therefore, this study aimed to reveal the mechanism of cytoskeletal proteins in epilepsy by investigating the expression of cytoskeletal proteins and synaptophysin (SYP) in mice at 0, 3, 6, and 24 h, 3 days, and 7 days in a kainic acid (KA)-induced epileptic model. Our results demonstrated that the expression of F-actin decreased significantly between 3 and 6 h, 6 and 24 h, and 24 h and 3 days (*P* < 0.05). Meanwhile, the expression of the neurofilament light chain, neurofilament medium chain, and neurofilament heavy chain subunits was significantly decreased (*P* < 0.001) at 3 h after the KA injection compared to the KA 0 h group, followed by an elevation at 6 h and a further decrease at 24 h compared to at 6 h. SYP expression was significantly decreased between 0 and 3 h as well as between 3 and 6 h (*P* < 0.05). At 24 h, the level was increased compared to at 6 h and continued to increase at 3 days after the KA injection. Thus, we propose that cytoskeletal proteins may be involved in the pathogenesis of epilepsy.

## 1 Introduction

Epilepsy is a significant cause of disability and death worldwide [1,2,3]. The World Health Organization has listed epilepsy as one of the five major global neurological and mental diseases and has advocated specific actions to solve the high burden of epilepsy on society [4,5]. Concerning the pathogenesis of epilepsy, it is known that it is related to neurotransmitters and ion channels, but its exact mechanisms are not well understood.

The neuronal cytoskeleton represents the basic structural framework for maintaining the morphology, structure, and biological function of neuronal cells [6,7]. The cytoskeleton of neurons, which includes microfilaments, microtubules, and neurofilaments (NFs, including the light chain, medium chain, and heavy chain subunits, NF-L, NF-M, NF-H, respectively), participates in maintaining neuronal cell stability; moreover, its dysregulation is involved in various nervous system diseases [8,9,10]. Specifically, the physiological structure, function, distribution, and abnormal expression of neuronal cytoskeletal proteins are linked to the occurrence and maintenance of epilepsy [11,12]. Importantly, the cytoskeleton has been shown to play a role in the process of neuronal synchronization during epilepsy [13,14]. In addition, synaptophysin (SYP) has been demonstrated to be a biomarker of synaptic transmission and synaptic reconstruction, which can trigger synaptic remodeling during epilepsy [14].

In this study, we aimed to reveal the mechanism of cytoskeletal proteins in epilepsy by analyzing the levels of cytoskeletal proteins and SYP during seizures in mice intraperitoneally injected with kainic acid (KA). Since cytoskeletal remodeling may contribute to the pathogenesis of epilepsy, it could potentially represent an unexplored therapeutic strategy for this debilitating disorder.

## 2 Materials and methods

### 2.1 Animals

Male and female adult C57BL/6 mice (weighing 20–22 g, aged 10 weeks old, and purchased from the Experimental Animal Center of Bethune Medical Department) had free access to food and drinking water and were housed at 25 ± 1°C with an alternating 12-h light/dark cycle.


**Ethical approval:** The research related to animal use has been complied with all the relevant national regulations and institutional policies for the care and use of animals. All animal experiments were carried out with the permission of The First Hospital, Jilin University (license number: 20210831) and in accordance with the Chinese laws for animal protection so that the number of animals used per experiment and their suffering were minimized.

### 2.2 Animal groups and drug administration

KA monohydrate (cat no. 58002-62-3) and sodium pentobarbital (cat no. 803-21-1) were purchased from Sigma-Aldrich (St. Louis, MO, USA). KA monohydrate was dissolved in 0.9% NaCl and administered intraperitoneally (30 mg/kg). The mice were divided into six groups of six animals each as follows: KA 0 h, KA 3 h, KA 6 h, KA 24 h, KA 3 days, and KA 7 days. The mortality rate of the mice was 30%. Sodium pentobarbital (Nembutal) (50 mg/kg) injected intraperitoneally was used as the anesthesia.

### 2.3 Behavioral studies

The behavior of the mice (three per experimental group) was studied by continuous observation after KA administration for 7 days. The Racine scale with minor modifications [15] was used to assess the seizure severity in the animals as follows: 0, behavioral arrest (motionless), hair raising, excitement, and rapid breathing; 1, movement of the mouth, lips, and tongue, vibrissae movement, and salivation; 2, head clonus and eye clonus; 3, forelimb clonus, “wet dog shakes”; 4, clonic rearing; 5, clonic rearing with loss of postural control and uncontrollable jumping.

### 2.4 Tissue preparation

All animals were decapitated following sodium pentobarbital anesthesia. Immediately after, the hippocampi of the brains were removed, frozen in liquid nitrogen, and stored at −80°C.

### 2.5 Western blotting

The whole hippocampal tissue lysates were prepared in radioimmunoprecipitation assay lysis buffer supplemented with Protease Inhibitor Cocktail and Phosphatase Inhibitor Cocktail. The total protein content was estimated by using a Pierce™ BCA Protein Assay Kit (Thermo Scientific), according to the manufacturer’s protocol. Equal amounts of proteins (60 μg per lane) from different groups (KA 0 h, KA 3 h, KA 6 h, KA 24 h, KA 3 days, and KA 7 days) were separated with BeyoGel™ SDS-PAGE Precast Gel and transferred onto a polyvinylidene difluoride membrane. The membranes were blocked for 30 min at room temperature in blocking buffer (5% skimmed milk in tris-buffered saline containing 0.1% Tween-20 (TBST)) and then probed overnight at 4°C with the appropriate primary antibody: mouse monoclonal anti-NF-H (1:100, Santa Cruz Biotechnology); mouse monoclonal anti-F-actin (1:500, Abcam); mouse monoclonal NF-L and NF-M (1:500, Santa Cruz Biotechnology); and mouse monoclonal anti-SYP and β-actin (1:2,000, Santa Cruz Biotechnology). β-actin was used as the loading control. The membrane was incubated with horseradish peroxidase-conjugated mouse IgGκ-binding protein (1:2,000; Santa Cruz Biotechnology) for 1 h at room temperature, followed by four washes with TBST. Images were captured using X-ray film (Fujifilm, Tokyo, Japan), and the band density was quantified using Image-Pro Plus (v. 6.0, Media Cybernetics, Silver Spring, MD, USA).

### 2.6 Histopathological staining

The paraffin sections were dewaxed with xylene (30 min for xylene I and 30 min for xylene II), dehydrated with an ethanol gradient (5 min for 100% ethanol I, 5 min for 100% ethanol II, 5 min for 95% ethanol, 5 min for 90% ethanol, and 5 min for 80% ethanol), washed with distilled water for 5 min (three times in total), soaked in hematoxylin for 10–20 min (paying attention to observe the color of the nucleus), immersed in hydrochloric acid–ethanol solution for 30 s for differentiation, washed with tap water several times, soaked in eosin dye solution for 10 min, rinsed with tap water, dehydrated and made transparent with alcohol, and sealed with neutral resin. Finally, the morphological changes of the hippocampus were observed under an optical microscope.

### 2.7 Statistical analysis

All western blot experiments were repeated three times. All statistical procedures were performed using the SPSS (18.0) statistical software package (IBM, New York, NY, USA). Data were represented as the mean ± standard deviation (SD). The comparisons between the control and experimental groups were performed using a one-way analysis of variance with a Tukey-Kramer post hoc test. The significance of all statistical comparisons was set at *P <* 0.05.

## 3 Results

### 3.1 Time-dependent seizure behavior of the epileptic mice

**Figure 1:**
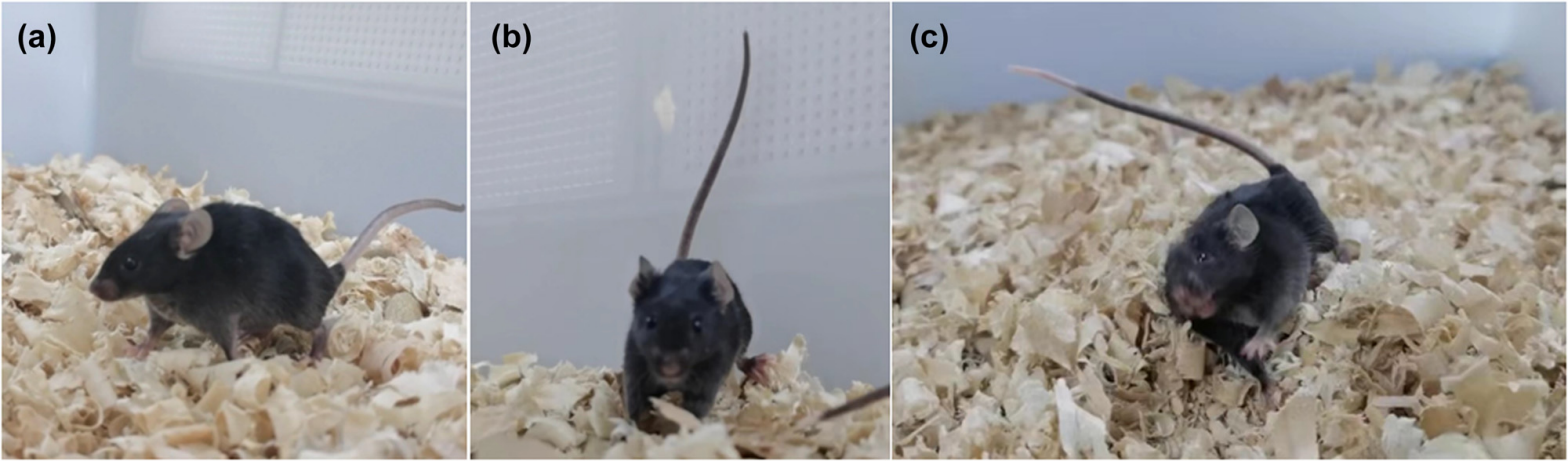
Time-dependent seizure behavior of the epileptic mice. The seizure behavior of the epileptic mice at 0–1 h (a), 2–3 h (b), and 4–6 h (c) after the KA injection.

The KA-induced seizure mouse model is extensively used in epilepsy research. In our study, we intraperitoneally injected KA into mice to induce seizure development. During the course of the experiment, the KA-injected mice were attributed a score of 0–5, according to the modified Racine scale (Table 1 and Figure 1). No seizure behavior was observed in the KA 0-h group (only normal activity and behavior). Injection of KA produced scores of 0, 1, and 2 during the first 1 h after administration; while scores of 3, 4, and 5 were recorded from 2–3 h. Scores of 4 and 5 were observed from 4 to 6 h postinjection. At 24 h, 3 days, and 7 days, scores of 4 and 5 were observed one to two times per day. In summary, the behavior assessment of the KA-injected mice was in line with the standards for an epilepsy model (Racine scores of 3, 4, and 5).

**Table 1:** Seizure behavior of KA-injected mice according to the modified Racine scale

Mouse number	Behavior score at time points after the KA injection		
	0–1 (h)	2–3 (h)	4–6 (h)
1	0/1/2	3/4/5	5
2	0/2	3/4/5	5
3	0/1/2	3/4/5	5
4	0/1/2	3/4	4/5
5	0/1	3/4	4/5
6	0/2	4/5	5
7	0/1/2	4/5	5
8	0/2	3/4/5	5
9	0/1/2	4/5	5
10	0/1/2	4/5	4/5
11	0/1	3/4	4/5
12	0/1/2	3/4/5	5
13	0/1/2	4/5	5
14	0/2	3/4/5	5
15	0/1/2	3/4/5	5
16	0/1/2	3/4	5
17	0/1/2	3/4	4/5
18	0/1/2	4/5	5

### 3.2 Dynamic expression of F-actin, NF-L, NF-M, and NF-H

**Figure 2:**
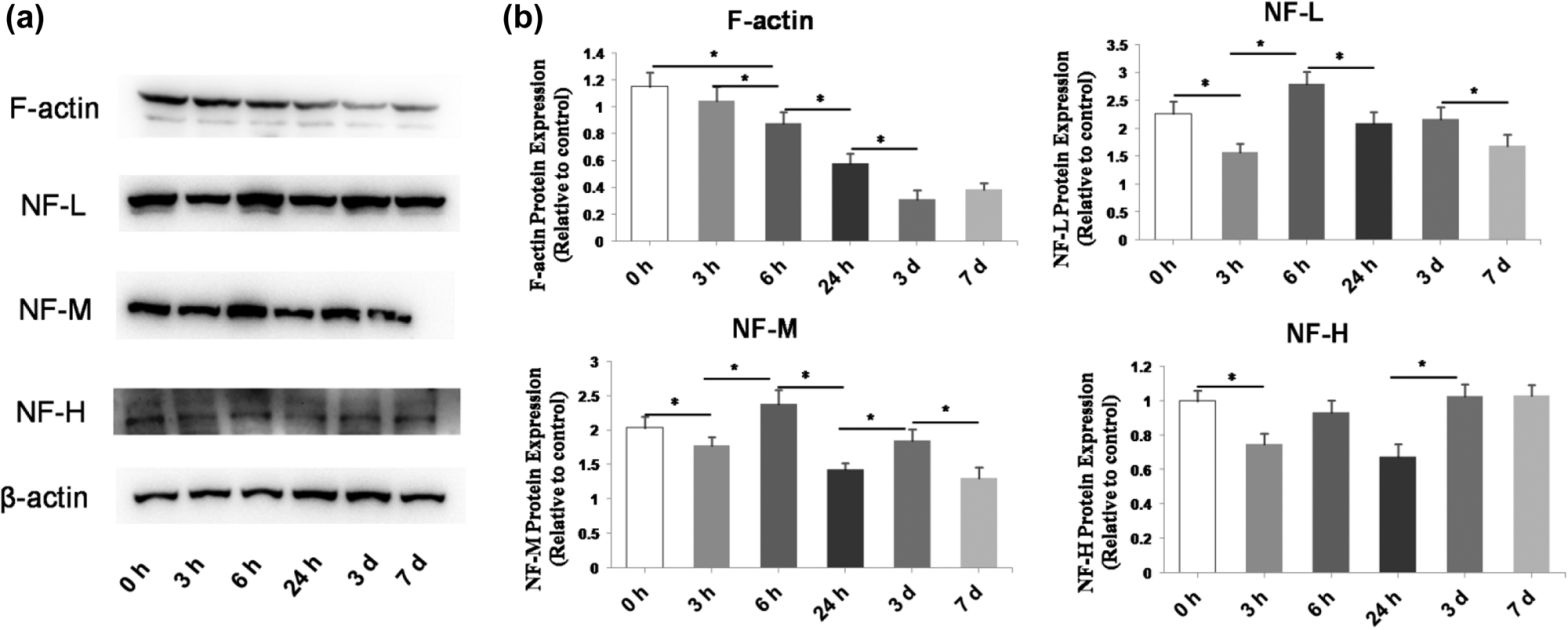
Time-dependent changes of the levels of F-actin, NF-L, NF-M, and NF-H in the brains of KA-injected mice. (a) Representative western blot images of protein bands. β-actin was used as a loading control. (b) Quantification of protein expression from the western blot images. The F-actin levels significantly decreased in a time-dependent manner. The levels varied and fluctuated over time. Data are presented as the mean ± SD. *P < 0.05.

To study the dynamic changes of microfilaments and NFs during the epileptic period, the expression levels of F-actin and the main components of NFs (NF-L, NF-M, and NF-H) were analyzed at different time points after the KA injection, i.e., 3, 6, and 24 h, 3 days, and 7 days, using western blotting (Figure 2a). We found that F-actin expression was significantly decreased between 3 and 6 h (*P* = 0.02), 6 and 24 h (*P* < 0.001), and 24 h and 3 days (*P* < 0.001). Meanwhile, the expression levels of NF-L, NF-M, and NF-H became significantly decreased (*P* < 0.001, *P* = 0.007, and *P* = 0.028, respectively) at 3 h after the KA injection compared to the KA 0 h group, followed by an elevation at 6 h and a further decrease at 24 h compared to at 6 h (Figure 2b).

### 3.3 Dynamic expression of SYP

**Figure 3:**
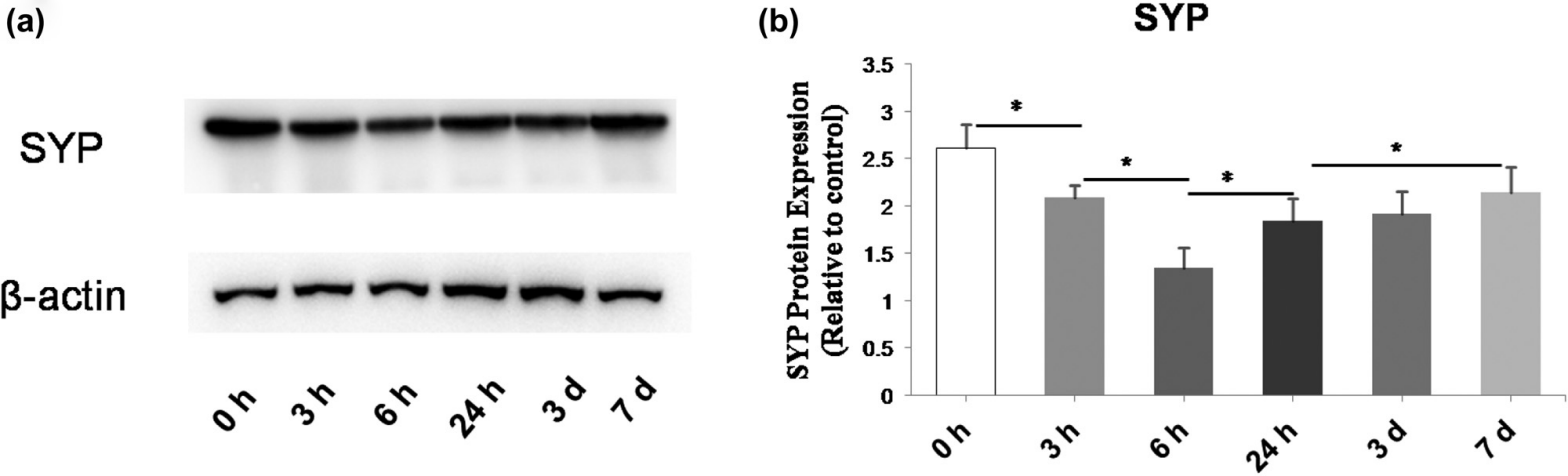
Time-dependent changes of SYP protein expression in the brains of KA-injected mice. (a) Representative western blot images of the protein bands. β-actin was used as a loading control. (b) Quantification of protein expression from the western blot images. The expression of SYP decreased at 6 h after the KA injection and then gradually increased until day 7. Data are presented as the mean ± SD. *P < 0.05.

The protein level of SYP was determined by western blot at 3, 6, 24 h, 3 days, and 7 days after KA administration (Figure 3a). We found that SYP expression was significantly decreased between 0 and 3 h (*P* = 0.002) as well as between 3 and 6 h (*P* < 0.001). At 24 h, the level was increased (compared to at 6 h; *P* = 0.003) and continued to increase at 3 days after the KA injection (Figure 3b).

### 3.4 Histopathological changes of the hippocampus in epileptic mice

**Figure 4:**
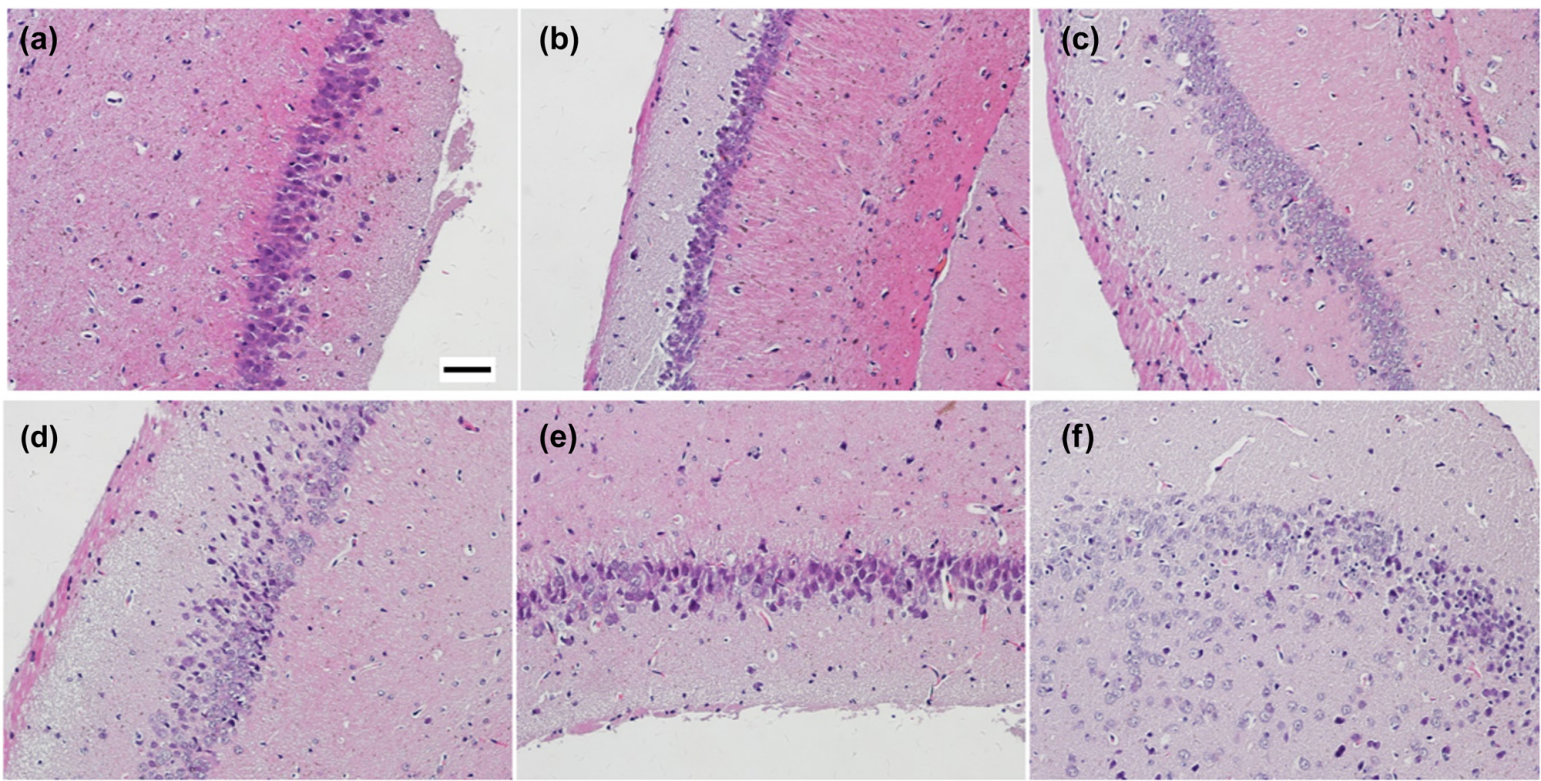
Histopathological changes of the hippocampus in epileptic mice. The time-dependent changes of hippocampal neurons at 0 h (a), 3 h (b), 6 h (c), 24 h (d), 3 days (e), and 7 days (f) after the KA injection.

The hippocampal neurons in the 0-h group had a normal morphology, were orderly arranged, and had a complete structure (Figure 4a). At 3 h after the KA injection, the neurons were mainly degenerated and showed interstitial edema (Figure 4b). At 6 h after the KA injection, the neurons were obviously lost or died (Figure 4c). At 24 h, 3 days, and 7 days after the KA injection, the lesion was further aggravated, with obvious neuronal loss, disordered arrangement of residual neurons, and proliferation of glial cells to different degrees (Figure 4d–f).

## 4 Discussion

This study aimed to investigate the time-dependent expression changes of cytoskeletal proteins and SYP in a KA mouse model. Our results demonstrated that F-actin expression was significantly decreased between 3 and 6 h, 6 and 24 h, and 24 h and 3 days. Meanwhile, the expression of NF-L, NF-M, and NF-H became significantly decreased at 3 h after the KA injection compared to the KA 0 h group, followed by an elevation at 6 h and a further decrease at 24 h compared to at 6 h. The initially decreased and subsequently increased expression of NFs was accompanied by a decrease of F-actin expression, which could result in the recombination of synapses. Meanwhile, SYP expression diminished and then gradually became elevated.

The pathogenesis of epilepsy is complex, involving many factors such as genetic predisposition, malformations, traumatic brain injury, chemical exposure, hypoxia, or stroke [16,17,18]. There are only a few studies reported in the literature regarding the pathogenesis of epilepsy focusing on the neuronal cytoskeleton. They have demonstrated that the irregular arrangement and winding of microtubules and microfilaments, synaptic reconstruction and remodeling, and the reduction of dendritic branches and spinous processes of neurons are the main pathological changes of the cytoskeletal components implicated in epilepsy [12,14].

Microfilaments are the smallest of the three neuronal cytoskeleton components and are mainly composed of actin. They are involved in the maintenance of neuronal cell morphology and the formation of tight junctions between cells [19,20]. Microfilaments exist in the form of globular actin or aggregated filamentous or fibrous actin (F-actin) [21]. It has been shown that the main morphological changes in the cerebral cortex during epilepsy are a decrease in dendritic components of pyramidal cells, especially dendritic spines, and a decrease in the number of axons and their terminals [22]. The decrease in the number of axons and their terminal dendritic spines is closely associated with the decrease of F-actin [23]. Our results demonstrated that the expression of F-actin in the brains of epileptic mice decreased significantly and continued to decline over time.

Alternatively, the significant decrease in F-actin expression suggests that microfilament depolymerization is necessary for the growth of neuronal axons [24]. A looser actin network is conducive to the extension of microtubules in the synaptic reconstruction process, but excessive microfilament depolymerization can cause its excessive growth. In addition, the increase of calcium influx and intracellular calcium overload can activate calpain, a calcium-dependent protease, which decomposes its substrates: actin and tubulin [25]. Activation of calpain by increased intracellular calcium can trigger activation of effector caspases and induce cell apoptosis. The neural cytoskeleton becomes disintegrated, leading to the excitatory injury of dendrites [26]. In addition, it has been reported that the density of dendritic spines in pyramidal cells of the hippocampus and neocortex is significantly decreased in the brain tissue of epileptic patients and animal models of focal epilepsy [27,28]. The reduction of dendritic spines is often accompanied by disarrangement of the dendritic part [29]. In the context of epilepsy, there is also the reconstruction of neural circuits [27]. Moreover, mossy fiber sprouting, which causes the formation of abnormal excitatory connections between nerve cells and increases excitatory sensitivity, contributes to seizure progression [24].

NFs are intermediate filaments found in neurons and axons that play important roles in maintaining the morphology of neurons and intercellular transmission [30]. The interactions among NFs, microtubules, microfilaments, and other cytoskeletal proteins regulate the diameter of axons and axoplasmic transport [31]. Therefore, the modifications of NFs can directly lead to changes in the shape and function of neurons and axons. Our results demonstrated that the expression of nerve filaments in the brains of epileptic mice significantly decreased in the beginning and then increased. We speculate that the excitatory injury occurred upon KA administration [23,24,25,26,27,28,29]. NFs exist in an unphosphorylated form in the perinuclear body and dendrites of neurons. Pathological phosphorylation of NFs may also result in a disorder of this neuronal arrangement. In addition, the aggregation of NFs and other cytoplasmic components in axons can affect the axoplasmic transport of various components, finally leading to neuronal death [32]. Furthermore, our results showed that the three different subunits of NFs did not change equally, which may have resulted in the disorganization of NFs.

SYP can be used as a molecular marker of the presynaptic vesicle membrane and also as a biomarker of synaptic transmission and synaptic reconstruction, which exists in almost all nerve terminals [33]. It has been previously reported that the synaptic density observed by electron microscopy was consistent with that measured by immunohistochemistry [33]. Therefore, as a reliable biomarker of nerve terminals, the localization and quantification of SYP-immunoreactive products can accurately reflect the distribution and density of synapses. SYP expression is elevated during axon sprouting, which leads to an increase in the number of presynaptic terminals and synaptic vesicles and hence the number of synapses, causing the occurrence of seizures [34]. Our results demonstrated that in the brains of epileptic mice, SYP expression was significantly decreased between 0 and 3 h as well as between 3 and 6 h. At 24 h, the level increased (compared to at 6 h) and continued to increase at 3 days after the KA injection. In fact, similar findings also have been discovered in the brain tissue of patients with temporal lobe epilepsy [33,34]. In addition, SYP participates in the release of Ca^2+^-dependent neurotransmitters (e.g., acetylcholine and glutamate), which regulate the release of endogenous glutamate in vesicles and affect synaptic plasticity [35]. Thus, the changes in the cytoskeleton and synaptic reconstruction after epilepsy are related to the development and persistence of KA-induced seizures.

## 5 Conclusions

In the current study, we demonstrated that different proteins involved in the composition of the cytoskeleton, such as F-actin, NF-L, NF-M, and NF-H, dynamically changed in the brains of KA-injected mice. Therefore, the cytoskeleton and its binding proteins, being the structural basis of neural network formation, may play an important role in the development of epilepsy. However, further research is still needed.
